# Geometric Neural Ordinary Differential Equations: From Manifolds to Lie Groups

**DOI:** 10.3390/e27080878

**Published:** 2025-08-19

**Authors:** Yannik P. Wotte, Federico Califano, Stefano Stramigioli

**Affiliations:** Robotics and Mechatronics, EEMCS, University of Twente (UT), Drienerlolaan 5, 7522 NB Enschede, The Netherlands

**Keywords:** neural ordinary differential equations, differential geometry, Lie groups, machine learning, optimal control

## Abstract

Neural ordinary differential equations (neural ODEs) are a well-established tool for optimizing the parameters of dynamical systems, with applications in image classification, optimal control, and physics learning. Although dynamical systems of interest often evolve on Lie groups and more general differentiable manifolds, theoretical results for neural ODEs are frequently phrased on $R^{n}$. We collect recent results for neural ODEs on manifolds and present a unifying derivation of various results that serves as a tutorial to extend existing methods to differentiable manifolds. We also extend the results to the recent class of neural ODEs on Lie groups, highlighting a non-trivial extension of manifold neural ODEs that exploits the Lie group structure.

## 1. Introduction

Ordinary differential equations (ODEs) are ubiquitous in the engineering sciences, from modeling and control of simple physical systems like pendulums and mass–spring–dampers, or more complicated robotic arms and drones, to the description of high-dimensional spatial discretizations of distributed systems, such as fluid flows, chemical reactions, or quantum oscillators. Neural ordinary differential equations (neural ODEs) [1,2] are ODEs parameterized by neural networks. Given a state *x*, and parameters θ representing the weights and biases of a neural network, a neural ODE reads as follows:

(1) $$\dot{x}=f_{\theta }\left(x,t\right)\;,x\left(0\right)=x_{0}\;.$$

First introduced by [1] as the continuum limit of recurrent neural networks, the number of applications of neural ODEs quickly exploded beyond simple classification tasks: learning highly nonlinear dynamics of multi-physical systems from sparse data [3,4,5], optimal control of nonlinear systems [6], medical imaging [7], and real-time handling of irregular time series [8], to name but a few. Discontinuous state transitions and dynamics [9,10], time-dependent parameters [11], augmented neural ODEs [12], and physics-preserving formulations [13,14] present further extensions that increase the expressivity of neural ODEs.

However, these methods are typically phrased for states $x\in R^{n}$. For many physical systems of interest, such as robot arms, humanoid robots, and drones, the state lives on differentiable manifolds and Lie groups [15,16]. More generally, the manifold hypothesis in machine learning raises the expectation that many high-dimensional data-sets evolve on intrinsically lower-dimensional, albeit more complicated, manifolds [17]. Neural ODEs on manifolds [18,19] presented significant steps to address this gap, with the first optimization methods for neural ODEs on manifolds. Yet, the general tools and approaches available on $R^{n}$, such as running costs, augmented states, time-dependent parameters, control inputs, or discontinuous state transitions, are rarely addressed in a manifold context. Similar issues persist in a Lie group context, where neural ODEs on Lie groups [20,21] have been formalized.

Our goal is to extend further architectures and costs for neural ODEs from $R^{n}$ to arbitrary manifolds (cf. Table 1), and in particular Lie groups, and to equip the reader with the technical background for their own extensions. Here the main conceptual challenge lies in phrasing chart-independent optimization methods [18,19] in a manner that easily adapts to a variety of neural ODE architectures and cost functions [1,3,12]. To this end we present a systematic approach for deriving geometric versions of the adjoint sensitivity method [1,2], which is a memory-efficient and scalable tool for the optimization of neural ODEs (cf. Section 1.1). Such benefits extend to manifolds and Lie groups [18,19,20,21]. A second challenge, both conceptual and practical, lies in expressing various manifolds in terms of local charts and in expressing neural-net-parameterized functions, dynamics, and tensor fields in local charts. To this end we classify existing methods into extrinsic [19,20] and intrinsic [18,21,22] approaches, a distinction inspired by well-known differential geometric concepts. In our context the distinction suggests different parameterizations, affects numerical integration techniques, and affects scaling to high-dimensional dynamics. Specifically, our contributions are as follows:

- Systematic derivation of adjoint methods for neural ODEs on manifolds and Lie groups, highlighting the differences and equivalence of various approaches—for an overview, see also Table 1;
- Summarizing the state of the art of manifold and Lie group neural ODEs by formalizing the notion of extrinsic and intrinsic neural ODEs;
- A tutorial on neural ODEs on manifolds and Lie groups, with a focus on the derivation of coordinate-agnostic adjoint methods for optimization of various neural ODE architectures. Readers will gain a conceptual understanding of the geometric nature of the underlying variables, a coordinate-free derivation of adjoint methods and learn to incorporate additional geometric and physical structures. On the practical side, this will aid in the derivation and implementation of adjoint methods with non-trivial terms for various architectures, also with regard to coordinate expressions and chart transformations.

The remainder of this article is organized as follows. A brief state of the art on neural ODEs concludes this introduction. Section 2 provides a background on differentiable manifolds, Lie groups, and the coordinate-free adjoint method. Section 3 describes neural ODEs on manifolds and derives parameter updates via the adjoint method for various common architectures and cost functions, including time-dependent parameters, augmented neural ODEs, running costs, and intermediate cost terms. Section 4 describes neural ODEs on matrix Lie groups, explaining the merits of treating Lie groups separately from general differentiable manifolds. Both Section 3 and Section 4 also classify methods into extrinsic and intrinsic approaches. We conclude with a discussion in Section 5, highlighting advantages, disadvantages, challenges, and promise of the presented material. Appendix A includes a background on Hamiltonian systems, which appear when transforming the adjoint method into a form that is unique to Lie groups.

### 1.1. Literature Review

For a general introduction to neural ODEs, see [25]. Neural ODEs on $R^{n}$ with fixed parameters were first introduced by [1], and parameter optimization via the adjoint method allowed for intermittent and final cost terms on each trajectory. The generalized adjoint method [2] also allows for running cost terms. Memory-efficient checkpointing is introduced in [26] to address stability issues of adjoint methods. Augmented neural ODEs [12] introduced augmented state spaces to allow neural ODEs to express arbitrary diffeomorphisms. Time-varying parameters were introduced by [11], with similar benefits to augmented neural ODEs. Neural ODEs with discrete transitions were formulated in [9,10], with [9] also learning event-triggered transitions common in engineering applications. Neural controlled differential equations (CDEs) were introduced in [27] for handling irregular time series, and parameter updates reapply the adjoint method [1]. Neural stochastic differential equations (SDEs) were introduced in [28], relying on a stochastic variant of the adjoint method for the parameter update. The previously mentioned literature phrases dynamics of neural ODEs on $R^{n}$.

Recent trends in research on neural ODEs focus on structure preservation to improve performance and reduce training time by appropriately restricting the class of parameterized vector fields. This includes symmetry preservation by equivariant [23] and approximately equivariant neural ODEs [29], which tackle symmetric and approximately symmetric time series and dynamics, e.g., in N-body dynamics and molecular dynamics. It also includes physics preservation in a physics learning context, where Hamiltonian neural networks [30,31] and (generalized) Lagrangian neural networks [32,33,34] improve performance by guaranteeing energy conservation. In control and model order reduction, port-Hamiltonian neural ODEs [3,35] further allow for learning models that interact with external ports in a power-preserving manner. These methods also phrase dynamics on $R^{n}$ and frequently apply the adjoint method for parameter updates.

Neural ODEs on manifolds were first introduced by [19], including an adjoint method on manifolds for final cost terms and application to continuous normalizing flows on Riemannian manifolds, but embedding manifolds into $R^{n}$. Neural ODEs on Riemannian manifolds are expressed in local exponential charts in [18], avoiding embedding into $R^{n}$ and considering final cost terms in the optimization. Charts for unknown, non-trivial latent manifolds together with dynamics in local charts are learned from high-dimensional data in [22], also including discretized solutions to partial differential equations. Parameterized equivariant neural ODEs on manifolds are constructed in [23], also commenting on state augmentation to express arbitrary (equivariant) flows on manifolds.

Neural ODEs on Lie groups were first introduced in [36] on the Lie group SE(3) to learn the port-Hamiltonian dynamics of a drone from an experiment, expressing group elements on an embedding $R^{12}$, and the approach was formalized to port-Hamiltonian systems on arbitrary matrix Lie groups in [20], embedding m×m matrices in $R^{m^{2}}$.

Neural ODEs on SE(3) were phrased in local exponential charts in [24] to optimize a controller for a rigid body using a chart-based adjoint method in local exponential charts. As an alternative, a Lie algebra-based adjoint method on general Lie groups was introduced in [21], foregoing Lie group-specific numerical issues of applying the adjoint method in local charts.

The choice of numerical solver in integrating neural ODEs and adjoint sensitivity equations is a nuanced area with much active research, especially for highly stiff [37], highly nonlinear [38,39], and structure-preserving neural ODEs [40]. We point towards the aforementioned sources for the interested reader. Results are expected to carry over into a manifold and Lie group context, where they hold in local charts. Also Lie group integrators [41,42] may be of interest for geometrically exact integration but are not well-investigated in a neural ODE context [20,21].

The optimization of neural ODEs via adjoint sensitivity methods is also referred to as “optimize-then-discretize” [25,43], since the formulation of the continuous adjoint system (called “optimize”) precedes their numerical solution (called “discretize”). This is opposed to “discretize-then-optimize” approaches, in which the neural ODE is first solved numerically (discretize) and gradients are then backpropagated through the numerical solver (optimize) [25,37,43]. Comparing the two, the constant memory efficiency of “optimize-then-discretize” approaches allows them to scale better to high-dimensional systems, giving them an edge for cases with more than 100 parameters and states [43]. Instead, “discretize-then-optimize” boasts higher accuracy and speed for low-dimensional systems, as well as highly stiff systems in which adjoint methods struggle with stability [37]. A popular discrete alternative to neural ODEs for physics-informed dynamics learning is given by variational integrator networks (VINs) [44,45], phrasing Lagrangian and Hamiltonian dynamics as discrete systems that conserve energy and the symplectic structure of the continuum dynamics [46,47]. Recent work [48] on Lie group forced VINs (LieFVINs) also allows inputs to the Lagrangian and Hamiltonian dynamics to be included in the variational formulation, allowing discrete optimal control. Both VINs and LieFVINs are applicable in a Lie group context, where they conserve geometry, symplecticity, and energy. The approach does not use adjoint methods for optimization and outperforms neural ODEs in the investigated conservative, low-dimensional dynamical systems [44,45,48]. Compared to continuous neural ODEs, both VINs and LieFVINs are discrete, which removes overhead from ODE solvers for lightweight applications, but their necessarily energy-based formulation presently restricts their use cases to conservative physical systems. We mention this promising area for completeness but narrow our attention to a geometric “optimize-then-discretize” approach via adjoint methods in the remainder of this article.

### 1.2. Notation

For a complete introduction to differential geometry see, e.g, [49], and for Lie group theory see [50].

Calligraphic letters M,N,… denote smooth manifolds. For conceptual clarity, the reader may think of these manifolds as embedded in a high-dimensional $R^{N}$, e.g., $M\subset R^{N}$. The set $C^{\infty }\left(M,N\right)$ contains smooth functions between M and N, and we define $C^{\infty }\left(M\right):=C^{\infty }\left(M,R\right)$.

The tangent space at x∈M is $T_{x}M$ and the cotangent space is $T_{x}^{*}M$. The tangent bundle of M is TM, and the cotangent bundle of M is $T^{*}M$. Then X(M) denotes the set of vector fields over M, and $\Omega^{k}\left(M\right)$ denotes the set of *k* forms, where $\Omega^{1}\left(M\right)$ are co-vector fields and $\Omega^{0}\left(M\right)=C^{\infty }\left(M\right)$ are smooth functions V:M→R. The exterior derivative is denoted as $d:\Omega^{k}\left(M\right)\to \Omega^{k+1}\left(M\right)$. For functions $V\in C^{\infty }\left(M\times N,R\right)$, with x∈M, y∈N, we denote by $\left(d_{x}V\right)\left(y\right)\in T_{x}^{*}M$ the partial differential at x∈M. Curves x:R→M are denoted as x(t), and their tangent vectors are denoted as $\dot{x}\in T_{x(t)}M$.

A Lie group is denoted by *G* and its elements by g,h. The group identity is e∈G, and *I* denotes the identity matrix. The Lie algebra of *G* is g, and its dual is $g^{*}$. Letters $\tilde{A},\tilde{B}$ denote vectors in the Lie algebra, while letters A,B denote vectors in $R^{n}$.

In coordinate expressions, lower indices are covariant and upper indices are contravariant components of tensors. For example for a (0,2)-tensor *M* the components $M_{ij}$ are covariant, and for non-degenerate *M* the components of its inverse $M^{-1}$ are $M^{ij}$, which are contravariant. We use the Einstein summation convention $a_{i}b^{i}:=\sum_{i}a_{i}b^{i}$; i.e., the product of variables with repeated lower and upper indices implies a sum.

Denoting *W* as a topological space, *D* the Borel σ-algebra, and P:D→[0,1] a probability measure, the tuple (W,D,P) denotes a probability space. Given a vector space *L* and a random variable C:X→L, the expectation of *C* with respect to P is $E_{w∼P}\left(C\right):=\int_{W}C\left(w\right)dP\left(w\right)$.

## 2. Background

### 2.1. Smooth Manifolds

Given an *n*-dimensional *manifold* M, with U⊂M being an open set and $Q:U\to R^{n}$ a homeomorphism, we call (U,Q) a *chart* and we denote the *coordinates* of x∈U as

(2) $$\left(q^{1},\ldots ,q^{n}\right):=Q\left(x\right),\;x\in U\subset M.$$

*Smooth* manifolds admit charts $\left(U_{1},Q_{1}\right)$ and $\left(U_{2},Q_{2}\right)$ with smooth *transition maps* $Q_{21}=Q_{2}\circ Q_{1}^{-1}$ defined on the intersection $U_{1}⋂U_{2}$, and a collection A of charts (U,Q) with smooth transition maps is called a smooth *atlas*. For examples of local charts for particular manifolds, see [49], Example 1.4, Example 1.5. A *vector field* f∈X(M) assigns a vector $f\left(x\right)\in T_{x}M$ at any point x∈M. This defines a dynamic system, also shown in a local chart (U,Q) with components $f^{i}\left(q\right),{\dot{q}}^{i}\in R$:

(3) $$\begin{array}{cccc} \dot{x} & =f\left(x\right)=f^{i}\left(q\right)\frac{\partial }{\partial Q^{i}}\;;\; & \; & x\left(0\right)=x_{0}\;, \end{array}$$

(4) $$\begin{array}{cccc} {\dot{q}}^{i} & =f^{i}\left(q\right)\;;\; & \; & q\left(0\right)=Q\left(x_{0}\right)\;. \end{array}$$

Solutions of (3) are then found by numerical integration of (4), applying chart transitions (e.g., $q_{2}\left(t\right)=Q_{21}\left(q_{1}\left(t\right)\right)$ from $q_{1}\left(t\right)=Q_{1}\left(x(t)\right)$ to $q_{2}\left(t\right)=Q_{2}\left(x(t)\right)$) during integration to avoid coordinate singularities (cf. Section 3.1.2). Denote the solution of (3) by the *flow operator*

(5) $$\Psi_{f}^{t}:M\to M\;;\;\Psi_{f}^{t}\left(x_{0}\right):=x\left(t\right)\;.$$

For a real-valued function $V\in C^{\infty }\left(M\right)$, its *differential* is the covector field

(6) $$dV\in \Omega^{1}\left(M\right)\;;\;dV=\frac{\partial V}{\partial q^{i}}dQ^{i}\;.$$

Additionally, given a smooth manifold N and a smooth map φ:N→M, with (U,Q) and $\left(\bar{U},\bar{Q}\right)$ appropriate charts of M and N, respectively, the *pullback* of dV via φ is

(7) $$\varphi^{*}dV\in \Omega^{1}\left(N\right)\;;\;\varphi^{*}dV:=d\left(V\circ \varphi \right)=\frac{\partial \varphi^{j}}{\partial {\bar{q}}^{i}}\frac{\partial V}{\partial q^{j}}d{\bar{Q}}^{i}\;.$$

With a *Riemannian metric M* (i.e., a symmetric, non-degenerate (0,2) tensor field) on M, the *gradient* of *V* is a uniquely defined vector field ∇V∈X(M) given by

(8) $$\nabla V:=M^{-1}dV=M^{ij}\frac{\partial V}{\partial q^{j}}\frac{\partial }{\partial q^{i}}\;.$$

When $M=R^{n}$, we assume that *M* is the Euclidean metric and pick coordinates such that the components of the gradient and differential are the same. Finally, we define the *Lie derivative* of 1-forms, which differentiates $\omega \in \Omega^{1}\left(M\right)$ along a vector field f∈X(M) and returns $L_{f}\omega \in \Omega^{1}\left(M\right)$:

(9) $$L_{f}\omega :=\frac{d}{dt}{\left({\Psi_{f}^{t}}^{*}\omega \right)}_{t=0}=\omega_{j}\left(\frac{\partial }{\partial q^{i}}f^{j}\right)dQ^{i}+\left(\frac{\partial }{\partial q^{j}}\omega_{i}\right)f^{j}dQ^{i}\;.$$

### 2.2. Lie Groups

Lie groups are smooth manifolds with a compatible group structure. We consider real matrix Lie groups G⊆GL(m,R), i.e., subgroups of the general linear group

(10) $$GL\left(m,R\right):=\{g\in R^{m\times m}\;|\;\det\left(g\right)\neq 0\}\;.$$

For g,h∈G the left and right translations by *h* are, respectively, the matrix multiplications

(11) $$\begin{array}{c} L_{h}\left(g\right):=hg\;, \end{array}$$

(12) $$\begin{array}{c} R_{h}\left(g\right):=gh\;. \end{array}$$

The Lie algebra of *G* is the vector space g⊆gl(m,R), with $\mathrm{gl}\left(m,R\right)=R^{m\times m}$ being the Lie algebra of GL(m,R).

Define a basis $E:=\{{\tilde{E}}_{1},\ldots ,{\tilde{E}}_{n}\}$ with ${\tilde{E}}_{i}\in g\subset R^{m\times m}$, and define the (invertible linear) map $\Lambda :R^{n}\to g$ as (equivalently (e.g, [51]), Λ and $\Lambda^{-1}$ are often denoted as the operators “hat” $\wedge :R^{n}\to R^{m\times m}$ and “vee” $\vee :R^{m\times m}\to R^{n}$, respectively)

(13) $$\Lambda :R^{n}\to g\;;\;\left(A^{1},\ldots ,A^{n}\right)\mapsto \sum_{i}A^{i}{\tilde{E}}_{i}\;.$$

The dual of g is denoted $g^{*}$, and given the map Λ we call $\Lambda^{*}:g^{*}\to R^{n}$ its dual. For $\tilde{A},\tilde{B}\in g$ the small adjoint ${\mathrm{ad}}_{\tilde{A}}\left(\tilde{B}\right)$ is a bilinear map, and the large adjoint ${\mathrm{Ad}}_{g}\left(\tilde{A}\right)$ is a linear map

(14) $$\begin{array}{cc} \mathrm{ad} & :g\times g\to g\;;\;{\mathrm{ad}}_{\tilde{A}}\left(\tilde{B}\right)=\tilde{A}\tilde{B}-\tilde{B}\tilde{A}\;, \end{array}$$

(15) $$\begin{array}{cc} \mathrm{Ad} & :G\times g\to g\;;\;{\mathrm{Ad}}_{g}\left(\tilde{A}\right)=g\tilde{A}g^{-1}\;. \end{array}$$

In the remainder of this article, we exclusively use the adjoint representation ${\mathrm{ad}}_{A}:R^{n}\to R^{n}$, written without a tilde in the subscript *A*, and adjoint representation ${\mathrm{Ad}}_{g}:R^{n}\times R^{n}$, which are obtained as

(16) $$\begin{array}{cc} {\mathrm{ad}}_{A} & :=\Lambda^{-1}\left({\mathrm{ad}}_{\Lambda (A)}\Lambda \left(\;\cdot \;\right)\right)\;, \end{array}$$

(17) $$\begin{array}{cc} {\mathrm{Ad}}_{g} & :=\Lambda^{-1}\left({\mathrm{Ad}}_{g}\Lambda \left(\;\cdot \;\right)\right)\;. \end{array}$$

The *exponential map* exp:g→G is a local diffeomorphism given by the matrix exponential ([50], Chapter 3.7)

(18) $$\exp\left(\tilde{A}\right):=\sum_{n=0}^{\infty }\frac{1}{n!}{\tilde{A}}^{n}\;.$$

Its inverse $\log:U_{\log}\to g$ is given by the matrix logarithm, and it is well-defined on a subset $U_{\log}\subset G$ ([50], Chapter 2.3):

(19) $$\log\left(g\right)=\sum_{n=1}^{\infty }{\left(-1\right)}^{n+1}\frac{{\left(g-I\right)}^{n}}{n}\;.$$

Often, these infinite sums in (18) and (19) can be further reduced to a finite sums in *m* terms by use of the Cayley–Hamilton theorem [52]. A chart $\left(U_{h},Q_{h}\right)$ on *G* that assigns zero coordinates to h∈G can be defined using (19) and (13):

(20) $$\begin{array}{cc} U_{h} & =\;\{hg\;|\;g\in U_{\log}\}\;, \end{array}$$

(21) $$\begin{array}{cc} Q_{h} & :\;U_{h}\to R^{n}\;;\;g\mapsto \Lambda^{-1}\log\left(h^{-1}g\right)\;, \end{array}$$

(22) $$\begin{array}{cc} Q_{h}^{-1} & :\;R^{n}\to G\;;\;q\mapsto h\exp\left(\Lambda (q)\right)\;. \end{array}$$

The chart $\left(U_{h},Q_{h}\right)$ is called an *exponential chart*, and a collection A of exponential charts $\left(U_{h},Q_{h}\right)$ that cover the manifold is called an *exponential atlas*.

The differential of a function $V\in C^{\infty }\left(G,R\right)$ is the co-vector field $dV\in \Omega^{1}\left(G\right)$ (see also Equation (6)). For any given g∈G we further transform the co-vector $dV\left(g\right)\in T_{g}^{*}G$ to a left-trivialized differential, which collects the components of the gradient expressed in $g^{*}$:

(23) $$\begin{array}{c} d_{g}^{L}V:=\Lambda^{*}L_{g}^{*}dV\left(g\right)=\frac{\partial }{\partial q}V{\left(g\left(I+\Lambda (q)\right)\right)}_{|q=0}\in R^{n}\;. \end{array}$$

For a derivation of this coordinate expression, see ([21], Section 3).

### 2.3. Gradient over a Flow

We are interested in computing the gradient of functions with respect to the initial state of a flow. The adjoint sensitivity equations are a set of differential equations that achieve this. In the following, we show a derivation of the adjoint sensitivity on manifolds ([21], App. A2). Given a function C:M→R, a vector field f∈X(M), the associated flow $\Psi_{f}^{t}:M\to M$, and a final time T∈R, the goal of the adjoint sensitivity method on manifolds is to compute the gradient

$$d\left(C\circ \Psi_{f}^{T}\right)\left(x_{0}\right)\;.$$

**Figure 1:**
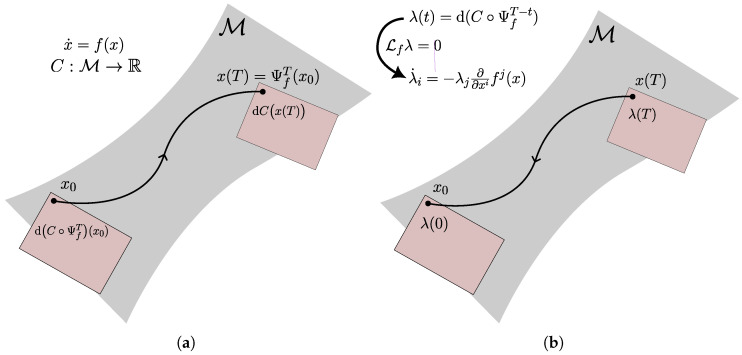
(**a**) The problem of computing the gradient over a flow, highlighting the cotangent spaces $dC\left(x(T)\right)\in T_{x(T)}^{*}M$ and $d\left(C\circ \Psi_{f}^{T}\right)\left(x_{0}\right)={\left(\Psi_{f}^{T}\right)}^{*}dC\left(x(T)\right)\in T_{x_{0}}^{*}M$. (**b**) In the adjoint method we set $\lambda \left(t\right)=d\left(C\circ \Psi^{T-t}\right)\left(x(t)\right)$, whose dynamics are uniquely determined by the property $L_{f}\lambda =0$, allowing us to find $\lambda \left(0\right)=d\left(C\circ \Psi_{f}^{T}\right)\left(x_{0}\right)$ by integrating $\dot{\lambda }$ backwards from $\lambda \left(T\right)=dC\left(x(T)\right)$.

In the adjoint method we define a co-state $\lambda \left(t\right)=d\left(C\circ \Psi^{T-t}\right)\left(x(t)\right)\in T_{x(t)}^{*}M$, which represents the differential of $C\left(x(T)\right)$ with respect to x(t). The adjoint sensitivity method describes its dynamics, which are integrated backwards in time from the known final condition $\lambda \left(T\right)=dC\left(x(T)\right)$, see also Figure 1. The adjoint sensitivity method is stated in Theorem 1.

**Theorem** **1** (Adjoint sensitivity on manifolds)**.** *The gradient of a function $C\circ \Psi_{f}^{T}$ is*

(24) $$d\left(C\circ \Psi_{f}^{T}\right)\left(x_{0}\right)=\lambda \left(0\right)\;,$$

where $\lambda \left(t\right)\in T_{x(t)}^{*}M$ is the co-state. In a local chart (U,Q) of M with coordinates q(t)=Q(x(t)), $\lambda \left(t\right)=\lambda_{i}\left(t\right)dQ^{i}$, the state and co-state satisfy the dynamics

(25) $$\begin{array}{cc} {\dot{q}}^{j} & =f^{j}\left(q\right)\;,\;q\left(0\right)=Q\left(x_{0}\right)\;, \end{array}$$

(26) $$\begin{array}{cc} {\dot{\lambda }}_{i} & =-\lambda_{j}\frac{\partial }{\partial q^{i}}f^{j}\left(q\right)\;,\;\lambda_{i}\left(T\right)=\frac{\partial C}{\partial q^{i}}\left(x(T)\right)\;. \end{array}$$

**Proof.**  Define the co-state $\lambda \left(t\right)\in T_{x(t)}^{*}M$ as

(27) $$\begin{array}{cc} \lambda (t) & :={\left(\Psi_{f}^{T-t}\right)}^{*}dC\left(x(T)\right)\;. \end{array}$$

Then Equation (24) is recovered by application of Equation (7):

(28) $$\lambda \left(0\right)={\left(\Psi_{f}^{T}\right)}^{*}dC\left(x(T)\right)=\left(dC\circ \Psi_{f}^{T}\right)\left(x_{0}\right)\;,$$

A derivation of the dynamics governing λ(t) constitutes the remainder of this proof. By definition of λ(t) and the Lie derivative (9), we have that $L_{f}\lambda \left(t\right)=0$:

(29) $$\begin{array}{cc} L_{f}\lambda \left(t\right)\; & =\frac{d}{ds}{\left({\left(\Psi_{f}^{s}\right)}^{*}\lambda \left(t+s\right)\right)}_{s=0}\\ \; & =\frac{d}{ds}\lambda \left(t\right)=0\;. \end{array}$$

If we further treat λ as a 1-form $\lambda \in \Omega^{1}\left(M\right)$ (denoted as λ by an abuse of notation), we obtain

$$\begin{array}{cc} L_{f}\lambda =\; & \lambda_{j}\left(\frac{\partial }{\partial q^{i}}f^{j}\right)dQ^{i}+\left(\frac{\partial }{\partial q^{j}}\lambda_{i}\right)f^{j}dQ^{i}=0\;. \end{array}$$

The components satisfy the partial differential equation

(30) $$\lambda_{j}\frac{\partial }{\partial q^{i}}f^{j}+f^{j}\frac{\partial }{\partial q^{j}}\lambda_{i}=0\;.$$

Impose that $\lambda \left(t\right)=\lambda \left(\Psi_{f}^{t}\left(x_{0}\right)\right)$ (this defines the 1-form λ along x(t)); then

(31) $${\dot{\lambda }}_{i}=\frac{\partial \lambda_{i}}{\partial q^{j}}{\dot{q}}^{j}=\frac{\partial \lambda_{i}}{\partial q^{j}}f^{j}\;.$$

Combining Equations (30) and (31) leads to Equation (26):

(32) $${\dot{\lambda }}_{i}=-\lambda_{j}\frac{\partial }{\partial q^{i}}f^{j}\;.$$

Expanding the final condition $\lambda \left(T\right)=dC\left(x(T)\right)$ in local coordinates (see Equation (6)) gives

(33) $$\lambda \left(T\right)=\frac{\partial C}{\partial q^{i}}\left(x(T)\right)dQ^{i}=\lambda_{i}\left(T\right)dQ^{i}\;\;\Leftrightarrow \;\lambda_{i}\left(T\right)=\frac{\partial C}{\partial q^{i}}\left(x(T)\right)\;.$$

□

Given a chart transition from a chart $\left(U_{1},Q_{1}\right)$ to a chart $\left(U_{2},Q_{2}\right)$, e.g., during numerical integration of (26), the respective co-state components $\lambda_{1,i}$ and $\lambda_{2,i}$ are related by a transformation $A_{i}^{j}=\partial_{i}\left(Q_{1}^{j}\circ Q_{2}^{-1}\right)$ as follows:

(34) $$\begin{array}{cc} \lambda_{i,2} & =A_{i}^{j}\lambda_{j,1}\;. \end{array}$$

A fact that will become useful in Section 4 is that Equations (25) and (26) have a Hamiltonian form. Define the control Hamiltonian $H_{c}:T^{*}M\to R$ as

(35) $$H_{c}\left(x,\lambda \right)=\lambda \left(f(x,t)\right)=\lambda_{i}\left(f^{i}\left(q,t\right)\right)\;.$$

Then Equation (25) and Equation (26), respectively, of Theorem 1 follow as the Hamiltonian equations on $T^{*}M$:

(36) $$\begin{array}{cc} {\dot{q}}^{j} & =\frac{\partial H_{c}}{\partial \lambda_{j}}=f^{j}\left(q,t\right)\;,\; \end{array}$$

(37) $$\begin{array}{cc} {\dot{\lambda }}_{i} & =-\frac{\partial H_{c}}{\partial q^{i}}=-\lambda_{j}\frac{\partial }{\partial q^{i}}f^{j}\left(q,t\right)\;. \end{array}$$

For a background on Hamilton’s equations, see also Appendix A.

## 3. Neural ODEs on Manifolds

A neural ODE on a manifold is an NN-parameterized vector field in X(M)—or including time dependence, it is an NN-parameterized vector field in X(M×R), with *t* in the R slot and $\dot{t}=1$. Given parameters $\theta \in R^{n_{\theta }}$, we denote this parameterized vector field as $f_{\theta }\left(x,t\right):=f\left(x,t,\theta \right)$. This results in the dynamic system

(38) $$\dot{x}=f_{\theta }\left(x,t\right)\;,\;x\left(0\right)=x_{0}\;.$$

The key idea of neural ODEs is to tackle various flow approximation tasks by optimizing the parameters with respect to a to-be-specified optimization problem. Denote a finite time horizon *T* and intermittent times $T_{1},T_{2},\ldots <T$. Denote a general trajectory cost by

(39) $$C_{f_{\theta }}^{T}\left(x_{0},\theta \right)=F\left(\theta ,\Psi_{f_{\theta }}^{T_{0}}\left(x_{0}\right),\Psi_{f_{\theta }}^{T_{1}}\left(x_{0}\right),\ldots ,\Psi_{f_{\theta }}^{T}\left(x_{0}\right)\right)+\int_{0}^{T}r\left(\Psi_{f_{\theta }}^{s}\left(x_{0}\right),s\right)ds\;,$$

with an intermittent and final cost term *F* and running cost *r*. Indicating a probability space (M,D,P), we define the total cost as

(40) $$J\left(\theta \right):=E_{x_{0}∼P}C_{f_{\theta }}^{T}\left(x_{0},\theta \right)\;.$$

The minimization problem takes the form

(41) $$\begin{array}{c} \min_{\theta }\;J\left(\theta \right)\;. \end{array}$$

Note that (41) is not subject to any dynamic constraint—the flow already appears explicitly in the cost $C_{f_{\theta }}^{T}$.

Normally, the optimization problem is solved by means of a stochastic gradient descent optimization algorithm [53]. In this, a batch of *N* initial conditions $x_{i}$ is sampled from the probability distribution corresponding to the probability measure P. Writing $C_{i}=C_{f_{\theta }}^{T}\left(x_{i},\theta \right)$, the parameter gradient $\frac{\partial }{\partial \theta }J\left(\theta \right)$ is approximated as

(42) $$\frac{\partial }{\partial \theta }J\left(\theta \right)=E_{x_{0}∼P}\frac{\partial }{\partial \theta }C_{f_{\theta }}^{T}\left(x_{0}\right)\approx \frac{1}{N}\sum_{i=0}^{N}\frac{\partial }{\partial \theta }C_{i}\;.$$

In this section, we show how to optimize the parameters θ for various choices of neural ODEs and cost functions, with (39) being the most general case of a cost, and highlight similarities in the various derivations. In the following, the gradient $\frac{\partial }{\partial \theta }C_{i}$ is computed via the adjoint method on manifolds for various scenarios. The advantage of the adjoint method over, e.g., automatic differentiation of $C_{i}$/backpropagation through an ODE solver is that it has a constant memory efficiency with respect to the network depth *T*.

### 3.1. Constant Parameters and Running and Final Cost

Here we consider neural ODEs of the form (38), with constant parameters θ and cost functions of the form

(43) $$C_{f_{\theta }}^{T}\left(x_{0},\theta \right)=F\left(\Psi_{f_{\theta }}^{T}\left(x_{0}\right),\theta \right)+\int_{0}^{T}r\left(\Psi_{f_{\theta }}^{s}\left(x_{0}\right),\theta ,s\right)ds\;,$$

with a final cost term *F* and a running cost term *r*. This generalizes [2] to manifolds. Compared to existing manifold methods for neural ODEs [18,54], the running cost is new.

The parameter gradient’s components $\frac{\partial }{\partial \theta }C_{f_{\theta }}^{T}\left((x_{0},t_{0}),\theta \right)\in R^{n_{\theta }}$ are then computed by Theorem 2 (see also [21]):

**Theorem** **2** (Generalized Adjoint Method on Manifolds)**.** *Given the dynamics* (38) *and the cost* (43)*, the parameter gradient’s components $\frac{\partial }{\partial \theta }C_{f_{\theta }}^{T}\left((x_{0},t_{0}),\theta \right)\in R^{n_{\theta }}$ are computed by*

(44) $$\frac{\partial }{\partial \theta }C_{f_{\theta }}^{T}\left((x_{0},t_{0}),\theta \right)=\left(\frac{\partial F}{\partial \theta }\right)\left(x\left(T\right),\theta \right)+\int_{0}^{T}\frac{\partial }{\partial \theta }\left(\lambda_{j}f_{\theta }^{j}\left(q\left(s\right)\right)+r\left(q\left(s\right),\theta ,s\right)\right)ds\;.$$

where the state x(s)∈M and co-state $\lambda \left(s\right)\in T_{x(s)}^{*}M$ satisfy, in a local chart (U,Q) with q(t)=Q(x(t)), $\lambda \left(t\right)=\lambda_{i}\left(t\right)dQ^{i}$,

(45) $$\begin{array}{cc} {\dot{q}}^{j} & =f_{\theta }^{j}\left(q,t\right)\;,\;q\left(0\right)=Q\left(x_{0}\right)\;,\;t\left(0\right)=t_{0}\;, \end{array}$$

(46) $$\begin{array}{cc} {\dot{\lambda }}_{i} & =-\lambda_{j}\frac{\partial }{\partial q^{i}}f_{\theta }^{j}\left(q,t\right)-\frac{\partial r}{\partial q^{i}}\;,\;\lambda_{i}\left(T\right)=\frac{\partial F}{\partial q^{i}}\left(x(T),\theta \right)\;. \end{array}$$

**Proof.**  Define the augmented state space as $M^{\prime }=M\times R^{n_{\theta }}\times R\times R$ to include the original state x∈M, parameters $\theta \in R^{n_{\theta }}$, accumulated running cost L∈R, and time t∈R in the augmented state $x^{\prime }:=\left(x,\theta ,L,t\right)\in M^{\prime }$. In addition, define the augmented dynamics $f_{\mathrm{aug}}\in X\left(M^{\prime }\right)$ as

(47) $${\dot{x}}^{\prime }=f_{\mathrm{aug}}\left(x^{\prime }\right)=\left(\begin{array}{c} f_{\theta }\left(x,t\right)\\ 0\\ r(x,\theta ,t)\\ 1 \end{array}\right)\;,\;x^{\prime }\left(0\right)=x_{0}^{\prime }:=\left(\begin{array}{c} x_{0}\\ \theta \\ 0\\ t_{0} \end{array}\right)\;.$$

This is an autonomous system with final state $x^{\prime }\left(T\right)=\left(x\left(T\right),\theta ,\int_{0}^{T}r\left(x,\theta ,s\right)ds,T\right)$. Next, define the cost $C_{\mathrm{aug}}:M^{\prime }\to R$ on the augmented space:

(48) $$C_{\mathrm{aug}}\left(x^{\prime }\right)=F\left(x,\theta \right)+L\;.$$

Then Equation (43) can be rewritten as the evaluation of a terminal cost $C_{\mathrm{aug}}\left(x^{\prime }\left(T\right)\right)$:

(49) $$C_{f_{\theta }}^{T}\left(x_{0}\right)=\left(C_{\mathrm{aug}}\circ \Psi_{f_{\mathrm{aug}}}^{T}\right)\left(x_{0}^{\prime }\right)\;.$$

By Theorem 1, the gradient $d\left(C_{\mathrm{aug}}\circ \Psi_{f_{\mathrm{aug}}}^{T}\right)$ is given by

(50) $$d\left(C_{\mathrm{aug}}\circ \Psi_{f_{\mathrm{aug}}}^{T}\right)\left(x_{0}^{\prime }\right)=\lambda \left(0\right)\;,$$

and by Equation (26), the components of λ(s) satisfy

(51) $$\begin{array}{c} {\dot{\lambda }}_{i}=-\lambda_{j}\frac{\partial }{\partial {q^{i}}^{\prime }}f_{\mathrm{aug}}^{j}\;,\;\lambda_{i}\left(T\right)=\frac{\partial }{\partial {q^{i}}^{\prime }}C_{\mathrm{aug}}\left(x^{\prime }\left(T\right)\right) \end{array}$$

Split the co-state into $\lambda_{q},\lambda_{\theta },\lambda_{L},\lambda_{t}$; then their components’ dynamics are as follows:

(52) $$\begin{array}{cc} {\dot{\lambda }}_{q,i} & =-\frac{\partial }{\partial q^{i}}\left(\lambda_{q,j}f_{\theta }^{j}\left(q,t\right)+\lambda_{L}r\left(q,\theta ,t\right)\right)\;,\;\lambda_{q}\left(T\right)=\frac{\partial F}{\partial q}\left(x\left(T\right),\theta \right)\;, \end{array}$$

(53) $$\begin{array}{cc} {\dot{\lambda }}_{\theta ,i} & =-\frac{\partial }{\partial \theta^{i}}\left(\lambda_{q,j}f_{\theta }^{j}\left(q,t\right)+\lambda_{L}r\left(q,\theta ,t\right)\right)\;,\;\lambda_{\theta }\left(T\right)=\frac{\partial F}{\partial \theta }\left(x\left(T\right),\theta \right)\;, \end{array}$$

(54) $$\begin{array}{cc} {\dot{\lambda }}_{L} & =0\;,\;\lambda_{L}\left(T\right)=\frac{\partial }{\partial L}C_{\mathrm{aug}}\left(x\left(T\right),\theta \right)=1\;, \end{array}$$

(55) $$\begin{array}{cc} {\dot{\lambda }}_{t} & =-\frac{\partial }{\partial t}\left(\lambda_{q,j}f_{\theta }^{j}\left(q,t\right)+\lambda_{L}r\left(q,\theta ,t\right)\right)\;,\;\lambda_{t}\left(T\right)=\frac{\partial }{\partial t}C_{\mathrm{aug}}\left(x\left(T\right),\theta \right)=0\;. \end{array}$$

The component $\lambda_{L}=1$ is constant, so Equation (52) coincides with (46). Integrating (53) from s=0 to s=T recovers Equation (44). $\lambda_{t}$ does not appear in any of the other equations, so Equation (55) may be ignored. □

In summary, the above proof depends on identifying a suitable augmented manifold $M^{\prime }$, with the goal that augmented dynamics $f_{\mathrm{aug}}\in X\left(M^{\prime }\right)$ are autonomous such that the cost function $C_{\mathrm{aug}}:M^{\prime }\to R$ on the augmented manifold rephrases the cost (43) as a final cost $C_{\mathrm{aug}}\left(x\left(T\right)\right)$. This allows Theorem 1 to be applied to derive the corresponding adjoint method. In later sections (Section 3.2), this process will be the main technical tool for generalizations of Theorem 2. The next sections describe common special cases of (38) and Theorem 2.

#### 3.1.1. Vanilla Neural ODEs and Extrinsic Neural ODEs on Manifolds

The case of neural ODEs on $R^{n}$ (e.g., [2]) is obtained by setting $M=R^{n}$. Scalar functions, vector fields, and tensor fields are readily expressed, see Table 2.

**Figure 2:**
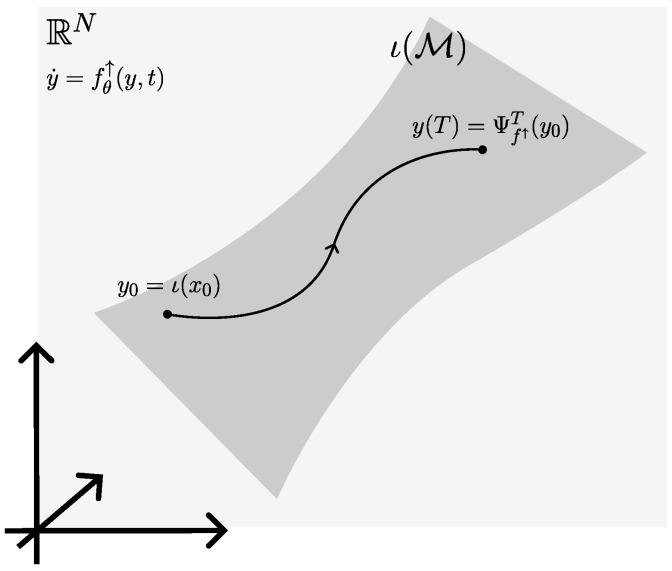
In the extrinsic formulation of neural ODEs on manifolds, the manifold M is embedded in $R^{N}$ as $\iota \left(M\right)\subset R^{N}$, and a neural ODE $f_{\theta }^{↑}\in X\left(R^{N}\right)$ is optimized.

There is an overlap with extrinsic neural ODEs on manifolds (described, for instance, in [19]), which optimize the neural ODE on an embedding space $R^{N}$, see also Figure 2.

We denote the embedding as $\iota :M\to R^{N}$, where x∈M and $y\in R^{N}$. Optimizing the neural ODE on $R^{N}$ requires extending the dynamics $f_{\theta }\left(\;\cdot \;,t\right)\in X\left(M\right)$ to a vector field $f_{\theta }^{↑}\left(\;\cdot \;,t\right)\in X\left(R^{N}\right)$ such that

(56) $$\iota_{*}f_{\theta }\left(x,t\right)=f_{\theta }^{↑}\left(\iota \left(x\right),t\right)\;.$$

The dynamics $f_{\theta }^{↑}\left(y,t\right)$ are then used in Theorem 2, and also the co-state lives in $T^{*}R^{N}$.

As shown in [19], the resulting parameter gradients are equivalent to those resulting from an application in local charts, as long as it can be guaranteed that the integral curves of $f^{↑}\left(y,t\right)$ remain within $\iota \left(M\right)\subset R^{N}$, i.e., are geometrically exact. Geometrically exact integration has to be guaranteed separately, either by integration in local charts [18] or stabilization techniques [55].

A strong upside of an extrinsic formulation is that existing neural ODE packages (e.g., [56]) can be applied directly. A downside to extrinsic neural ODEs is that finding $f^{↑}\left(y,t\right)$ may not be immediate, since tangency to ι(M) is required, see also Table 2. Finally, the extrinsic dimension *N* can be much larger than the intrinsic dimension n=dimM, leading to computational overhead that does not fully exploit the manifold hypothesis. Extrinsic methods for neural ODEs are the preferred choice when the intrinsic dimension *n* is small and there is a known embedding $\iota \left(M\right)\subset R^{N}$ with low extrinsic dimension *N*. Then the computational overhead due to N>n is negligible, and stabilization techniques [55] can be applied to guarantee geometrically exact integration.

#### 3.1.2. Intrinsic Neural ODEs on Manifolds

**Figure 3:**
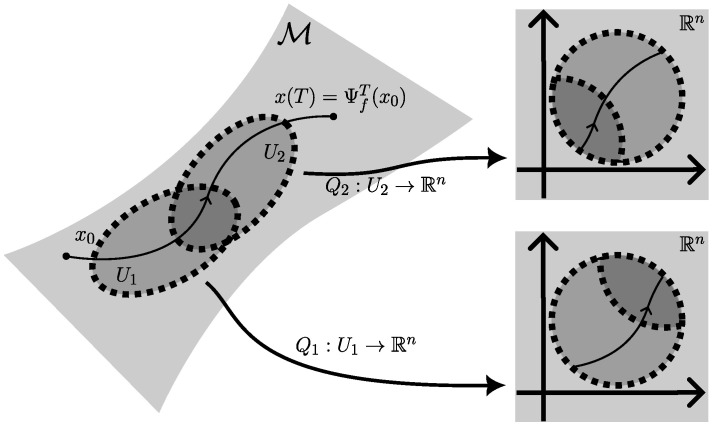
In the intrinsic formulation of neural ODEs on manifolds, the neural ODE $f_{\theta }\in X\left(M\right)$ is optimized in local charts, here $\left(U_{1},Q_{1}\right)$ and $\left(U_{2},Q_{2}\right)$, and the state and co-state undergo chart transitions.

The intrinsic case of neural ODEs on manifolds [18] is described by integrating the dynamics in local charts, see also Figure 3.

The advantage of intrinsic over extrinsic neural ODEs on manifolds is that the dimension of the resulting equations is as low as possible in the intrinsic case for a given manifold. The flexibility of chart representations gives intrinsic neural ODEs on manifolds the power to represent high-dimensional data distributions at their latent dimension, see especially [22] for learning charts from data and [18,21] for chart-switching methods during numerical integration. Numerical integration in local charts is also geometrically exact by default. However parameterization of scalar functions, vector fields, and tensor fields with neural networks in local charts, as well as their differentiation with respect to parameters, presents a source of complexity. There are three common methods to parameterize scalar-valued functions $V\in C^{\infty }\left(M\right)$ in local charts (vector fields and tensor-valued functions directly follow by parameterizing their scalar component functions in an analogous way):

- A partition of unity $\sigma_{i}:R^{n}\to R$ with respect to a collection of charts $\left(U_{i},Q_{i}\right)$ can be used to sum over chart -components $V_{i}:R^{n}\to R$ as $V\left(x\right):=\sum_{i}\sigma_{i}\left(Q_{i}\left(x\right)\right)V_{i}\left(Q_{i}\left(x\right)\right)$, see examples in [49], Chapter 2, and [24].
- The function *V* can be directly defined by chart representatives $V_{i}:=V\circ Q_{i}^{-1}$, enforcing compatibility between overlapping charts $\left(U_{i},Q_{i}\right),\left(U_{j},Q_{j}\right)$ by soft constraints, which are implemented as additional cost terms $∥V_{i}\left(q_{i}\right)-V_{j}\circ Q_{j}\circ Q_{i}^{-1}\left(q_{i}\right)∥$ that are minimized on chart overlaps $U_{i}\cap U_{j}$, see [18,22].
- Given an embedding $\iota :M\to R^{N}$ and $\bar{V}\in C^{\infty }\left(R^{N}\right)$, an extrinsic representation $V_{i}:=\bar{V}\circ \iota \circ Q_{i}^{-1}$ is possible, see [19,21].

Advantages and disadvantages are summarized in Table 3.

In available state-of-the-art packages for neural ODEs, the chart dynamics are phrased as discontinuous dynamics with state transitions $Q_{1}\circ Q_{2}^{-1}:R^{n}\to R^{n}$, but implementation is not yet streamlined for local charts, chart transitions of the co-state (cf. Section 2.3), and custom adjoint sensitivity equations.

#### 3.1.3. Structure Preservation

Structure-preserving architectures narrow down the class of learnable neural ODEs from arbitrary vector fields X(M) to subsets of X(M) with particular properties, improving training speed and performance (cf. Section 1.1). Examples of such subsets are (symmetry-preserving) equivariant dynamical systems [23] and (physics-preserving) Hamiltonian, Lagrangian, and port-Hamiltonian dynamical systems [3]. Given that a structure-preserving parameterization of the neural ODE is known in closed form, these are readily implemented in the above formalism.

For example, reusing results from Table 2 and Table 3, Hamiltonian and Lagrangian neural ODEs [30,32] are fully determined by scalar functions $H_{\theta }\in C^{\infty }\left(T^{*}Q\right),L_{\theta }\in C^{\infty }\left(TQ\right)$, respectively, and their gradients. Hamiltonian neural ODEs are advantageous for joint learning of the dynamics and energy of conservative physical systems, where the learned Hamiltonian vector fields $X_{H_{\theta }}\left(T^{*}Q\right)$ are guaranteed to conserve the Hamiltonian $H_{\theta }$ representing the total energy. Lagrangian neural ODEs likewise enable learning the dynamics of conservative physical systems and enable incorporation of dissipative terms [34] but do not directly represent the total energy.

Port-Hamiltonian neural ODEs [3,35] offer further expressiveness: besides a scalar Hamiltonian $H_{\theta }\in C^{\infty }\left(M\right)$, they offer degrees of freedom in a skew-symmetric (2,0)-tensor $J_{\theta }$ (called a Poisson tensor), a positive-definite (2,0)-tensor $R_{\theta }$ (called a dissipation tensor), and a linear input map $B_{\theta }\left(x\right):R^{k}\to T_{x}M$. This allows learning the dynamics of non-conservative dynamical systems that can be coupled with known physical systems and control inputs through the input map, while jointly learning the total energy $H_{\theta }$, rate of energy dissipation $R_{\theta }\left(dH_{\theta },dH_{\theta }\right)$, and externally supplied power (see [57] for an introductory overview). Most physical systems can be represented in a port-Hamiltonian form [58], giving this parametrization a high degree of expressiveness that has been used in dynamics learning [3], control [20,21], and model order reduction [35]. Albeit not investigated in practice, this expressiveness may also be a disadvantage compared to Lagrangian or Hamiltonian neural networks, resulting in overfitting when, e.g., small dissipation terms are learned where there is no dissipation. Generally speaking, choosing the most specific structure-preserving neural network is advised.

### 3.2. Extensions

The proof of Theorem 2 depended on identifying a suitable augmented manifold $M^{\prime }$, autonomous augmented dynamics $f_{\mathrm{aug}}\in X\left(M^{\prime }\right)$, and an augmented cost function $C_{\mathrm{aug}}:M^{\prime }\to R$ that rephrases the cost (43) as a final cost $C_{\mathrm{aug}}\left(x\left(T\right)\right)$ to apply Theorem 1. This approach generalizes to various other scenarios, including different cost terms, augmented neural ODEs on manifolds, and time-dependent parameters, presented in the following.

#### 3.2.1. Nonlinear and Intermittent Cost Terms

We consider here the case of neural ODEs on manifolds of the form (38) with cost (39). This is a generalization of [1], in which intermittent cost terms appear for neural ODEs on $R^{n}$. For the final and intermittent cost term $F_{\theta }:M\times M\times \ldots \times M\to R$, we denote by $d_{k}F_{\theta }\in T_{x}^{*}M$ the differential with respect to the *k*-th slot and denote θ as a subscript to avoid confusion. The components of $d_{k}F$ will be denoted $\frac{\partial F}{\partial^{k}q^{i}}$. In this case, the parameter gradient is determined by repeated application of Theorem 2:

**Theorem** **3** (Generalized Adjoint Method on Manifolds)**.** *Given the dynamics (38) and the cost (39), the parameter gradient’s components $\frac{\partial }{\partial \theta }C_{f_{\theta }}^{T}\left((x_{0},t_{0}),\theta \right)\in R^{n_{\theta }}$ are computed by*

(57) $$\begin{array}{cc} \frac{\partial }{\partial \theta }C_{f_{\theta }}^{T}\left((x_{0},t_{0}),\theta \right)= & \left(\frac{\partial F}{\partial \theta }\right)\left(\theta ,x\left(T_{1}\right),x\left(T_{2}\right),\ldots ,x\left(T\right)\right)\\ \; & +\int_{0}^{T}\frac{\partial }{\partial \theta }\left(\lambda_{j}f_{\theta }^{j}\left(q\left(s\right)\right)+r\left(q\left(s\right),\theta ,s\right)\right)ds\;. \end{array}$$

where the state x(s)∈M satisfies (45) and the co-state $\lambda \left(s\right)\in T_{x(s)}^{*}M$ satisfies dynamics with discrete updates at times $T_{1},\ldots ,T_{N-1}$ given by

(58) $$\begin{array}{cc} {\dot{\lambda }}_{q,i} & =\frac{\partial }{\partial q^{i}}\left(\lambda_{q,j}f_{\theta }^{j}\left(q,t\right)+r\left(q,\theta ,t\right)\right)\;;\;\lambda_{q,i}\left(T\right)=\frac{\partial F_{\theta }}{\partial^{N}q^{i}}\left(x\left(T_{1}\right),\ldots ,x\left(T\right)\right) \end{array}$$

(59) $$\begin{array}{cc} \lambda_{i}\left(T_{k,-}\right) & =\lambda_{i}\left(T_{k,+}\right)+\frac{\partial F_{\theta }}{\partial^{k}q^{i}}\left(x\left(T_{1}\right),\ldots ,x\left(T\right)\right)\;, \end{array}$$

*with $T_{k,-}$ being the instance after a discrete update at time $T_{k}$ (recall that co-state dynamics are integrated backwards, so $T_{k,-}<T_{k}<T_{k,+}$) and $T_{k,+}$ the instance before.*

**Proof.**  We introduce an augmented manifold $M^{\prime }=M\times \ldots \times M\times R^{n_{\theta }}\times R\times R$ to include *N* copies of the original state x∈M, parameters $\theta \in R^{n_{\theta }}$, accumulated running cost L∈R, and time t∈R in the augmented state $x^{\prime }:=\left(x_{1},\ldots ,x_{N},\theta ,L,t\right)\in M^{\prime }$. Let

(60) $$\varrho_{T_{i}}\left(t\right)=\left\{\begin{array}{c} 1\;t\leq T_{i}\\ 0\;t>T_{i} \end{array}\right.\;,$$

and define the augmented dynamics $f_{\mathrm{aug}}\in X\left(M^{\prime }\right)$ as

(61) $${\dot{x}}^{\prime }=f_{\mathrm{aug}}\left(x^{\prime }\right)=\left(\begin{array}{c} \varrho_{T_{1}}\left(t\right)f_{\theta }\left(x_{1},t\right)\\ \vdots \\ \varrho_{T_{N-1}}\left(t\right)f_{\theta }\left(x_{N-1},t\right)\\ f_{\theta }\left(x_{N},t\right)\\ 0\\ r(x_{N},\theta ,t)\\ 1 \end{array}\right)\;,\;x^{\prime }\left(0\right)=x_{0}^{\prime }:=\left(\begin{array}{c} x_{0}\\ \vdots \\ x_{0}\\ x_{0}\\ \theta \\ 0\\ t_{0} \end{array}\right)\;.$$

This is an autonomous system with final state

(62) $$x^{\prime }\left(T\right)=\left(x\left(T_{1}\right),\ldots ,x\left(T_{N-1}\right),x\left(T\right),\theta ,\int_{0}^{T}r\left(x,\theta ,s\right)ds,T\right)\;.$$

Next, define the cost $C_{\mathrm{aug}}:M^{\prime }\to R$ on the augmented space:

(63) $$C_{\mathrm{aug}}\left(x^{\prime }\right)=F_{\theta }\left(x_{1},\ldots ,x_{N}\right)+L\;.$$

Then Equation (39) can be rewritten as the evaluation of a terminal cost $C_{\mathrm{aug}}\left(x^{\prime }\left(T\right)\right)$:

(64) $$C_{f_{\theta }}^{T}\left(x_{0}\right)=\left(C_{\mathrm{aug}}\circ \Psi_{f_{\mathrm{aug}}}^{T}\right)\left(x_{0}^{\prime }\right)\;.$$

Apply Equation (26), and split the co-state into $\lambda_{q_{1}},\ldots ,\lambda_{q_{N}},\lambda_{\theta },\lambda_{L},\lambda_{t}$; then their components’ dynamics are as follows:

(65) $$\begin{array}{cc} {\dot{\lambda }}_{q_{1},i} & =-\frac{\partial }{\partial q^{i}}\left(\lambda_{q_{1},j}\varrho_{T_{1}}\left(t\right)f_{\theta }^{j}\left(q_{1},t\right)\right)\;,\;\lambda_{q_{1}}\left(T\right)=\frac{\partial F_{\theta }}{\partial^{1}q}\left(x\left(T_{1}\right),\ldots ,x\left(T\right)\right)\;,\\ \vdots \end{array}$$

(66) $$\begin{array}{cc} {\dot{\lambda }}_{q_{N},i} & =-\frac{\partial }{\partial^{N}q^{i}}\left(\lambda_{q_{N},j}f_{\theta }^{j}\left(q_{N},t\right)+\lambda_{L}r\left(q_{N},\theta ,t\right)\right)\;,\;\lambda_{q_{N}}\left(T\right)=\frac{\partial F_{\theta }}{\partial^{N}q}\left(x\left(T_{1}\right),\ldots ,x\left(T\right)\right)\;, \end{array}$$

(67) $$\begin{array}{cc} {\dot{\lambda }}_{\theta ,i} & =-\frac{\partial }{\partial \theta^{i}}\left(\lambda_{q_{1},j}\varrho_{T_{1}}\left(t\right)f_{\theta }^{j}\left(q_{1},t\right)+\ldots +\lambda_{q_{N},j}f_{\theta }^{j}\left(q_{N},t\right)+\lambda_{L}r\left(q,\theta ,t\right)\right)\;,\\ \lambda_{\theta }\left(T\right) & =\frac{\partial F_{\theta }}{\partial \theta }\left(x\left(T_{1}\right),\ldots ,x\left(T\right)\right)\;. \end{array}$$

We excluded the dynamics of $\lambda_{t}$, which does not appear in any of the other equations, and the constant $\lambda_{L}=1$. Finally, define the cumulative co-state

(68) $$\lambda_{q}=\varrho_{T_{1}}\left(t\right)\lambda_{q_{1}}+\ldots +\lambda_{q_{N}}\;.$$

Its dynamics at $t\in \left[0,T\right]\;T_{1},\;\cdot \;,T_{N-1}$ are given by the sum of (65) to (66), letting $q=q_{N}$:

(69) $$\begin{array}{cc} {\dot{\lambda }}_{q,i} & ={\dot{\lambda }}_{q_{1},i}+\ldots +{\dot{\lambda }}_{q_{N},i} \end{array}$$

(70) $$\begin{array}{cc} \; & =\frac{\partial }{\partial q^{i}}\left(\lambda_{q,j}f_{\theta }^{j}\left(q,t\right)+r\left(q,\theta ,t\right)\right) \end{array}$$

(71) $$\begin{array}{cc} \lambda_{q}\left(T\right) & =\frac{\partial F_{\theta }}{\partial^{N}q}\left(x\left(T_{1}\right),\ldots ,x\left(T\right)\right)\;, \end{array}$$

with discrete jumps (58) accounting for the final conditions of $\lambda_{q_{1}},\ldots ,\lambda_{q_{N}}$, and the dynamics (67) can be rewritten as

(72) $${\dot{\lambda }}_{\theta ,i}=\frac{\partial }{\partial \theta^{i}}\left(\lambda_{q,j}f_{\theta }^{j}\left(q,t\right)+r\left(q,\theta ,t\right)\right)\;;\;\lambda_{\theta }\left(T\right)=\frac{\partial F_{\theta }}{\partial \theta }\left(x\left(T_{1}\right),\ldots ,x\left(T\right)\right)\;.$$

Integrating this from s=0 to s=T recovers Equation (57). □

Cost terms of this form are interesting for optimization of, e.g., periodic orbits [59] or trajectories on manifolds, where conditions at multiple checkpoints $\Psi_{f_{\theta }}^{T_{i}}\left(x_{0}\right)$ may appear in the cost.

#### 3.2.2. Augmented Neural ODEs on Manifolds and Time-Dependent Parameters

With state x∈M, augmented state α∈N (not to be confused with $x^{\prime }\in M^{\prime }$), and parameterized $\varphi_{\theta }:M\to N$, augmented neural ODEs on manifolds are neural ODEs on the manifold M×N of the form

(73) $$\left(\begin{array}{c} \dot{x}\\ \dot{\alpha } \end{array}\right)=\left(\begin{array}{c} f_{\theta }\left(x,\alpha \right)\\ g_{\theta }\left(x,\alpha \right) \end{array}\right)\;;\;\left(\begin{array}{c} x(0)\\ \alpha (0) \end{array}\right)=\left(\begin{array}{c} x_{0}\\ \varphi_{\theta }\left(x_{0}\right) \end{array}\right)\;.$$

Time *t* is not included explicitly in these dynamics, since it can be included in α. This case also includes the scenario of time-dependent parameters $\bar{\theta }\left(t\right)$ as part of α. As the trajectory cost, we take a final cost

(74) $$C_{f_{\theta },g_{\theta }}^{T}\left(x_{0},\theta \right)=F\left(\Psi_{f_{\theta },g_{\theta }}^{T}\left(x_{0},\varphi_{\theta }\left(x_{0}\right)\right),\theta \right)\;.$$

This is a generalization of [11,12].

**Theorem** **4** (Adjoint Method for Augmented Neural ODEs on Manifolds)**.** *Given the dynamics* (73) *and the cost *(74)*, the parameter gradient’s components $\frac{\partial }{\partial \theta }C_{f_{\theta }}^{T}\left((x_{0},t_{0}),\theta \right)\in R^{n_{\theta }}$ are computed by*

(75) $$\begin{array}{cc} \frac{\partial }{\partial \theta }C_{f_{\theta },g_{\theta }}^{T}\left((x_{0},\varphi \left(x_{0}\right)),\theta \right)= & \left(\frac{\partial F}{\partial \theta }\right)\left(x\left(T\right),\alpha \left(T\right),\theta \right)+\frac{\partial \varphi^{j}}{\partial \theta }\lambda_{\alpha ,j}\left(0\right)\\ \; & +\int_{0}^{T}\frac{\partial }{\partial \theta }\left(\lambda_{x,j}f_{\theta }^{j}\left(q\left(s\right)\right)+\lambda_{\alpha ,j}g_{\theta }^{j}\left(q\left(s\right)\right)\right)ds\;. \end{array}$$

where the states x(s)∈M,α(s)∈N satisfy (73) and co-states $\lambda_{x}\left(s\right)\in T_{x(s)}^{*}M,\lambda_{\alpha }\left(s\right)\in T_{\alpha (s)}^{*}N,$ satisfy, in a local chart (U,Q) on M and $\bar{U},\bar{Q}$ on N,

(76) $$\begin{array}{cc} {\dot{\lambda }}_{x,i} & =-\frac{\partial }{\partial q^{i}}\left(\lambda_{x,j}f_{\theta }^{j}\left(q,\bar{q},t\right)+\lambda_{\alpha ,j}g_{\theta }^{j}\left(q,\bar{q},t\right)\right)\;,\;\lambda_{x,i}\left(T\right)=\frac{\partial F}{\partial q^{i}}\left(x(T),\alpha (T),\theta \right)\;, \end{array}$$

(77) $$\begin{array}{cc} {\dot{\lambda }}_{\alpha ,i} & =-\frac{\partial }{\partial {\bar{q}}^{i}}\left(\lambda_{x,j}f_{\theta }^{j}\left(q,\bar{q},t\right)+\lambda_{\alpha ,j}g_{\theta }^{j}\left(q,\bar{q},t\right)\right)\;,\;\lambda_{\alpha ,i}\left(T\right)=\frac{\partial F}{\partial {\bar{q}}^{i}}\left(x(T),\alpha (T),\theta \right)\;. \end{array}$$

**Proof.**  Define the augmented state space as $M^{\prime }=M\times N\times R^{n_{\theta }}$ to include the states x∈M,α(s)∈N and parameters $\theta \in R^{n_{\theta }}$ in the augmented state $x^{\prime }:=\left(x,\alpha ,\theta \right)\in M^{\prime }$. In addition, define the augmented dynamics $f_{\mathrm{aug}}\in X\left(M^{\prime }\right)$ as

(78) $${\dot{x}}^{\prime }=f_{\mathrm{aug}}\left(x^{\prime }\right)=\left(\begin{array}{c} f_{\theta }\left(x,\alpha \right)\\ g_{\theta }\left(x,\alpha \right)\\ 0 \end{array}\right)\;,\;x^{\prime }\left(0\right)=x_{0}^{\prime }:=\left(\begin{array}{c} x_{0}\\ \varphi_{\theta }\left(x_{0}\right)\\ \theta \end{array}\right)\;.$$

This is an autonomous system with final state $x^{\prime }\left(T\right)=\left(x\left(T\right),\alpha \left(T\right),\theta \right)$. Next, define the cost $C_{\mathrm{aug}}:M^{\prime }\to R$ on the augmented space:

(79) $$C_{\mathrm{aug}}\left(x^{\prime }\right)=F\left(x,\alpha ,\theta \right)\;.$$

Then Equation (43) can be rewritten as the evaluation of a terminal cost $C_{\mathrm{aug}}\left(x^{\prime }\left(T\right)\right)$. The gradient $d\left(C_{\mathrm{aug}}\circ \Psi_{f_{\mathrm{aug}}}^{T}\right)$ is given by an application of Equation (26). Split the co-state into $\lambda_{x},\lambda_{\alpha },\lambda_{\theta }$; then their components’ dynamics are as follows:

(80) $$\begin{array}{cc} {\dot{\lambda }}_{x,i} & =-\frac{\partial }{\partial q^{i}}\left(\lambda_{x,j}f_{\theta }^{j}\left(q,\bar{q},t\right)+\lambda_{\alpha ,j}g_{\theta }^{j}\left(q,\bar{q},t\right)\right)\;,\;\lambda_{x}\left(T\right)=\frac{\partial F}{\partial q}\left(x\left(T\right),\alpha \left(T\right),\theta \right)\;, \end{array}$$

(81) $$\begin{array}{cc} {\dot{\lambda }}_{\alpha ,i} & =-\frac{\partial }{\partial {\bar{q}}^{i}}\left(\lambda_{x,j}f_{\theta }^{j}\left(q,\bar{q},t\right)+\lambda_{\alpha ,j}g_{\theta }^{j}\left(q,\bar{q},t\right)\right)\;,\;\lambda_{\alpha ,i}\left(T\right)=\frac{\partial F}{\partial {\bar{q}}^{i}}\left(x(T),\alpha (T),\theta \right)\;, \end{array}$$

(82) $$\begin{array}{cc} {\dot{\lambda }}_{\theta ,i} & =-\frac{\partial }{\partial \theta^{i}}(\lambda_{x,j}f_{\theta }^{j}\left(q,\bar{q},t\right)+\lambda_{\alpha ,j}g_{\theta }^{j}\left(q,\bar{q},t\right))\;,\;\lambda_{\theta }\left(T\right)=\frac{\partial F}{\partial \theta }\left(x\left(T\right),\alpha \left(T\right),\theta \right)\;. \end{array}$$

Since $\alpha \left(0\right)=\varphi_{\theta }\left(x_{0}\right)$ also depends on θ, the total gradient of the cost with respect to θ is given by

(83) $$\frac{\partial }{\partial \theta^{i}}C_{f_{\theta }}^{T}\left((x_{0},\varphi_{\theta }\left(x_{0}\right)),\theta \right)=\lambda_{\theta ,i}\left(0\right)+\frac{\partial \varphi^{j}}{\partial \theta^{i}}\lambda_{\alpha ,j}\left(0\right)\;.$$

Integrate (82) to find $\lambda_{\theta ,i}\left(0\right)$; then Equation (75) is recovered. □

Augmented neural ODEs are universal function approximators ([25], Chapter 2). Potential applications of augmented neural ODEs on manifolds include, e.g., the optimization of guiding vector fields for path-following of closed or self-intersecting paths [60], where state augmentation sits at the core of formulating singularity-free guiding vector fields for self-intersecting paths. In the same context, discontinuous initializations $g_{\alpha }\left(x_{0}\right)$ allow globally stabilizing controllers to be represented for topologically non-trivial manifolds (e.g., the sphere $S^{2}$), where smooth controllers are necessarily not globally stable. A further degenerate application of Theorem 4 is obtained by removing *x*, i.e., fixing x=0 and $f_{\theta }\left(x,\alpha \right)=0$ in Equation (73). Then both the dynamics $g_{\theta }\left(\alpha \right)$ and initial condition $\alpha \left(0\right)=\varphi_{\theta }\left(0\right)$ are parameterized by θ, allowing joint optimization of the parameters and initial condition. This is interesting for joint optimization and numerical continuation, e.g., [59].

## 4. Neural ODEs on Lie Groups

Just as a neural ODE on a manifold is an NN-parameterized vector field in X(M) (or, including time, X(M×R)), a neural ODE on a Lie group can be seen as a parameterized vector field in X(G) (or X(G×R)). Similarly to Equation (38), this results in a dynamic system

(84) $$\dot{g}=f_{\theta }\left(g,t\right)\;,\;g\left(0\right)=g_{0}\;.$$

Yet, Lie groups offer more structure than manifolds: the Lie algebra g provides a canonical space to represent tangent vectors, and its dual $g^{*}$ provides a canonical space to represent the co-state. Similarly, canonical (exponential) charts offer a structure for integrating dynamic systems [41]. Frequently, dynamics on a Lie group induce dynamics on a manifold M: by means of an action

(85) Φ:G×M→M;(g,x)↦Φ(g,x),

evolutions g(t) induce evolutions $x\left(t\right)=\Phi (g\left(t\right),x_{0})$ on M. This makes neural ODEs on Lie groups interesting in their own right.

In this section, we describe optimizing (41) for the cost

(86) $$C_{f_{\theta }}^{T}\left(g_{0},\theta \right)=F\left(\Psi_{f_{\theta }}^{T}\left(g_{0}\right),\theta \right)+\int_{0}^{T}r\left(\Psi_{f_{\theta }}^{s}\left(g_{0}\right),\theta ,s\right)ds\;,$$

with a final cost term *F* and a running cost term *r*. We highlight the extrinsic approach and two intrinsic approaches, where one of the latter is particular to Lie groups.

### 4.1. Extrinsic Neural ODEs on Lie Groups

The extrinsic formulation of neural ODEs on Lie groups was first introduced by [20] and applies ideas of [54] (see also Section 3.1.1). Given G⊂GL(m,R), this formulation treats the dynamic system (84) as a dynamic system on $R^{m^{2}}$. Denote $\mathrm{vec}:R^{m\times m}\to R^{m^{2}}$ as an invertible map that stacks the components of an input matrix into a component vector (in canonical coordinates on $R^{m\times m}$ and $R^{m^{2}}$, though this choice is not required.) and let ${\mathrm{proj}}_{G}:R^{m\times m}\to G$ be a projection onto $G\subset R^{m\times m}$. Further denote $A_{y}={\mathrm{vec}}^{-1}\left(y\right)$ and $g_{y}={\mathrm{proj}}_{G}\left(A_{y}\right)$. A lift $f_{\theta }^{↑}\left(y,t\right)$ can then be defined as

(87) $$f_{\theta }^{↑}\left(y,t\right)=\mathrm{vec}\left(A_{y}g_{y}^{-1}f\left(g_{y},\theta ,t\right)\right)\;.$$

As was the case for extrinsic neural ODEs on manifolds, the cost gradient resulting from this optimization is well-defined and equivalent to any intrinsically defined procedure. However, the dimension $m^{2}$ of the vectorization can be significantly larger than the intrinsic dimension of the Lie group.

### 4.2. Intrinsic Neural ODEs on Lie Groups

Theorem 2 directly applies to optimization of neural ODEs on Lie groups, given the local exponential charts (20) and (21) on *G*. This does not make full use of the available structure on Lie groups. Frequently, dynamical systems are of a left-invariant form (88) or a right-invariant form (89)

(88) $$\begin{array}{cc} \dot{g} & =g\Lambda \left(\rho_{\theta }^{L}\left(g,t\right)\right)\;, \end{array}$$

(89) $$\begin{array}{cc} \dot{g} & =\Lambda \left(\rho_{\theta }^{R}\left(g,t\right)\right)g\;. \end{array}$$

Denote $K\left(q\right):T_{q}R^{n}\to R^{n}$ as the derivative of the exponential map (see [21] for details). Then the chart representatives $f_{\theta }^{i}$ in a local exponential chart $\left(U_{h},Q_{h}\right)$ are

(90) $$\begin{array}{cc} f_{\theta }^{L,i}\left(q,t\right) & ={\left(K^{-1}\right)}_{j}^{i}\left(q\right)\rho^{L,j}\left(Q_{h}^{-1}\left(q\right)\right)\;, \end{array}$$

(91) $$\begin{array}{cc} f_{\theta }^{R,i}\left(q,t\right) & ={\left(K^{-1}\right)}_{j}^{i}\left(q\right){\mathrm{Ad}}_{Q_{h}^{-1}\left(q\right)}\rho^{R,j}\left(Q_{h}^{-1}\left(q\right)\right)\;. \end{array}$$

Application of Theorem 2 then requires computing $\frac{\partial }{\partial q^{j}}f_{\theta }^{L,i}\left(q,t\right)$ or $\frac{\partial }{\partial q^{j}}f_{\theta }^{R,i}\left(q,t\right)$. But this leads to significant computational overhead due to differentiation of the terms ${\left(K^{-1}\right)}_{j}^{i}\left(q\right)$ (see [21]). Instead of applying Theorem 2, i.e., expressing dynamics in local charts, the dynamics can also be expressed in the Lie algebra g. Theorem 1 has a Hamiltonian form, which can be directly transformed into Hamiltonian equations on a Lie group (see also Appendix A). Applying this reasoning to Theorem 2, we arrive at the following form, which foregoes differentiating ${\left(K^{-1}\right)}_{j}^{i}\left(x\right)$:

**Theorem** **5** (Left Generalized Adjoint Method on Matrix Lie Groups)**.** *Given the dynamics* (88) *and the cost* (86)*, or the dynamics* (89) *with $\rho_{\theta }^{L}\left(g,t\right)={\mathrm{Ad}}_{g^{-1}}\rho_{\theta }^{R}\left(g,t\right)$, the parameter gradient $\frac{\partial }{\partial \theta }C_{f_{\theta }}^{T}\left(g_{0}\right)$ of the cost is given by the integral equation*

(92) $$\frac{\partial }{\partial \theta }C_{f_{\theta }}^{T}\left(g_{0}\right)=\frac{\partial F}{\partial \theta }\left(g\left(T\right),\theta \right)+\int_{0}^{T}\frac{\partial }{\partial \theta }\left(\lambda_{g}^{⊤}\rho_{\theta }^{L}\left(g,s\right)+r\left(g,\theta ,s\right)\right)ds\;,$$

*where the state g(t)∈G and co-state $\lambda_{g}\left(t\right)\in R^{n}$ are the solutions of the system of equations*

(93) $$\begin{array}{cc} \dot{g} & =f_{\theta }\left(g,t\right)\;,\;g\left(0\right)=g_{0}\;, \end{array}$$

(94) $$\begin{array}{cc} {\dot{\lambda }}_{g} & =-d_{g}^{L}\left(\lambda_{g}^{⊤}\rho_{\theta }^{L}\left(g,s\right)+r\left(g,\theta ,s\right)\right)+{\mathrm{ad}}_{\rho_{\theta }^{L}\left(g,t\right)}^{⊤}\lambda_{g}\;,\;\lambda_{g}\left(T\right)=d_{g}^{L}F\left(g\left(T\right),\theta \right)\;. \end{array}$$

**Proof.**  This is proven in two steps. First, define the time- and parameter-dependent control Hamiltonian $H_{c}:T^{*}M\times R^{n_{\theta }}\times R\to R$ as

(95) $$H_{c}\left(x,\lambda ,\theta ,t\right)=\lambda \left(f_{\theta }\left(x,t\right)\right)+r\left(x,\theta ,t\right)=\lambda_{i}\left(f_{\theta }^{i}\left(q,t\right)\right)+r\left(q,\theta ,t\right)\;.$$

The equations for the state and co-state dynamics (45) and (46), respectively, of Theorem 2 follow as the Hamiltonian equations on $T^{*}M$:

(96) $$\begin{array}{cc} {\dot{q}}^{j} & =\frac{\partial H_{c}}{\partial \lambda_{j}}=f_{\theta }^{j}\left(q,t\right)\;,\; \end{array}$$

(97) $$\begin{array}{cc} {\dot{\lambda }}_{i} & =-\frac{\partial H_{c}}{\partial q^{i}}=-\lambda_{j}\frac{\partial }{\partial q^{i}}f_{\theta }^{j}\left(q,t\right)-\frac{\partial r}{\partial q^{i}}\;. \end{array}$$

And the integral Equation (44) reads

(98) $$\frac{\partial }{\partial \theta }C_{f_{\theta }}^{T}\left((x_{0},t_{0}),\theta \right)=\frac{\partial F}{\partial \theta }\left(x\left(T\right),\theta \right)+\int_{0}^{T}\frac{\partial H_{c}}{\partial \theta }dt\;.$$

Second, rewrite the control Hamiltonian (95) on a Lie group *G*, i.e., $H_{c}:T^{*}G\times \times R^{n_{\theta }}\times R\to R$. By substituting $\lambda_{g}\left(t\right)=\Lambda^{*}L_{g}^{*}\lambda \left(t\right)$ (see also Equation (A6)), this induces $H_{c}:G\times g^{*}\times R^{n_{\theta }}\times R\to R$,

(99) $$H_{c}\left(g,\lambda_{g},\theta ,t\right)=\lambda_{g}^{⊤}\rho_{\theta }^{L}\left(g,t\right)+r\left(g,\theta ,t\right)\;.$$

Finally Hamilton’s equations (96) and (97) are rewritten in their form on a matrix Lie group by means of (A7) and (A8), which recovers Equations (93) and (94):

(100) $$\begin{array}{cc} \dot{g} & =g\Lambda (\frac{\partial H_{c}}{\partial \lambda_{g}})\;, \end{array}$$

(101) $$\begin{array}{cc} {\dot{\lambda }}_{g} & =-d_{g}^{L}H_{c}+{\mathrm{ad}}_{\frac{\partial H_{c}}{\partial \lambda_{g}}}^{⊤}\lambda_{g}\;. \end{array}$$

To find the final condition for $\lambda_{g}$, use that $\lambda_{g}\left(t\right)=\Lambda^{*}L_{g}^{*}\lambda \left(t\right)$:

(102) $$\lambda_{g}\left(T\right)=\Lambda^{*}L_{g}^{*}\lambda \left(T\right)=\Lambda^{*}L_{g}^{*}dF\left(g\left(T\right),\theta \right)=d_{g}^{L}F\left(g\left(T\right),\theta \right)\;.$$

□

Similar equations also hold on abstract (non-matrix) Lie groups, see [21]. Compared to the extrinsic method of Section 4.1, Theorem 5 has the advantage that the dimension of the co-state $\lambda_{g}$ is as low as possible. Compared to the chart-based approach on Lie groups, Theorem 5 foregoes differentiating through the terms $K_{j}^{i}\left(q\right)$, avoiding overhead. Compared to a chart-based approach on manifolds, the choice of charts is also canonical on Lie groups. Although the Lie group approach foregoes many of the pitfalls of intrinsic neural ODEs on manifolds, implementation in existing neural ODE packages is currently cumbersome: the adjoint sensitivity equations (94) have a non-standard form, requiring an adapted dynamics of the co-state λ, but these equations are rarely intended for modification in existing packages. Packages for geometry-preserving integrators on Lie groups, such as [41], are also not readily available for arbitrary Lie groups.

### 4.3. Extensions

The proof of Theorem 5 relied on finding a control Hamiltonian formulation for Theorem 2. This approach generalizes to methods in Section 3.2, which rely on the use of Theorem 1. This is because Theorem 1 itself has a Hamiltonian form ([21,54]).

A further straightforward extension of the methods presented in this Section are port-Hamiltonian neural ODEs on Lie groups [20]. In [20], these are systems with a configuration on a Lie group *G* and momentum on $g^{*}$. In terms of the theory presented above, such port-Hamiltonian dynamics can be phrased as a dynamic system on a product Lie group $G\times g^{*}$ (taking vector addition as the group operation on $g^{*}$), recovering both extrinsic [20] and intrinsic [21] port-Hamiltonian neural ODEs on Lie groups.

## 5. Discussion

We discuss advantages and disadvantages of the main flavors of the presented formulations for manifold neural ODEs, expanding on the previous sections. We focus on extrinsic (embedding dynamics in $R^{N}$) and intrinsic (integrating in local charts) formulations. The prior comments can be summarized as follows:

- The extrinsic formulation is readily implemented if the low-dimensional manifold M and an embedding into $R^{N}$ are known. This comes at the possible cost of geometric inexactness and a higher dimension of the co-state and sensitivity equations.
- The co-state in the intrinsic formulation has a generally lower dimension, which reduces the dimension of the sensitivity equations. The chart-based formulation also guarantees geometrically exact integration of dynamics. This comes at the mild cost of having to define local charts and chart-transitions.

This dimensionality reduction is unlikely to have a high impact when the manifold M is known and low-dimensional, e.g., for the sphere $M=S^{2}$ or similar manifolds. However, when applying the manifold hypothesis to high-dimensional data, there might be non-trivial latent manifolds for which the embedding is not immediate and where the latent manifold is of a much lower dimension than the embedding data manifold. Then the intrinsic method becomes difficult to avoid. If geometric exactness of the integration is desired, local charts also need to be defined for the extrinsic approach, in which case the intrinsic approach may offer further advantages.

In order to derive neural ODEs on Lie groups, three approaches are possible: the extrinsic and intrinsic formulations on manifolds directly carry over to matrix Lie groups, embedding G⊂GL(m,R) in $R^{m^{2}}$ or using local exponential charts, respectively. A third option is a novel intrinsic method for neural ODEs on matrix Lie groups, which makes full use of the Lie group structure by phrasing dynamics on g (as is more common on Lie groups) and the co-state on $g^{*}$, avoiding difficulties of the chart-based formalism in differentiating extra terms.

Prior comments on advantages and disadvantages of these flavors can be summarized as follows:

- The extrinsic formulation on matrix Lie groups can come at much higher cost than that on manifolds, since the intrinsic dimension of *G* can be much lower than $m^{2}$ and a higher dimension of the co-state and sensitivity equations can be obtained. Geometrically exact integration procedures are more readily available for matrix Lie groups, integrating $\dot{g}$ in local exponential charts.
- The chart-based formulation on matrix Lie groups struggles when dynamics are not naturally phrased in local charts. This is common; dynamics are often more naturally phrased on g. This was alleviated by an algebra-based formulation on matrix Lie groups. Both are intrinsic approaches that feature co-state dynamics that are as low as possible. However, the algebra-based approach still lacks readily available software implementation.

The authors believe that the algebra-based formulation is more convenient in principle and consider software implementations of the algebra-based approach as possible future work.

In summary, we presented a unified, geometric approach to extend various methods for neural ODEs on $R^{N}$ to neural ODEs on manifolds and Lie groups. Optimization of neural ODEs on manifolds was based on the adjoint method on manifolds. Given a novel cost function *C* and neural ODE architecture *f*, the strategy to present the results in a unified fashion was to identify a suitable augmented manifold $M_{\mathrm{aug}}$, augmented dynamics $f_{\mathrm{aug}}\in X\left(M_{\mathrm{aug}}\right)$, and cost $C_{\mathrm{aug}}:M_{\mathrm{aug}}\to R$ such that the original cost function can be rephrased as $C=C_{\mathrm{aug}}\circ \Psi_{f_{\mathrm{aug}}}^{T}$. To further derive optimization of intrinsic neural ODEs on Lie groups, we found a Hamiltonian formulation of the adjoint method on manifolds and subsequently transformed it into Hamiltonian equations on a matrix Lie group.

## Figures and Tables

**Table 1:** Summary of neural ODEs on manifolds and Lie groups presented in this article.

Name of Neural ODE	Subtype	Trajectory Cost	Subsection	Originally Introduced in
Neural ODEs on manifolds (Section 3)	Extrinsic	Running and final cost	Section 3.1.1	Final cost [19], running cost [21]
	Intrinsic	Running and final cost, intermittent cost	Section 3.1.2 and Section 3.2.1	Final cost [18], running cost [21], intermittent cost (this work)
	Augmented, time-dependent parameters	Final cost	Section 3.2.2	Augmenting M to TM [23], Augmenting M to M×N (this work)
Neural ODEs on Lie groups (Section 4)	Extrinsic	Final cost and intermittent cost	Section 4.1	In [20]
	Intrinsic, dynamics in local charts	Running and final cost	Section 4.2	In [21,24]
	Intrinsic, dynamics on Lie algebra	Running and final cost	Section 4.2	In [21]

**Table 2:** Parameterization of functions in extrinsic neural ODEs.

Function	Vanilla Neural ODE	Extrinsic Neural ODE
Scalar fields $V_{\theta }\left(x\right)\in R$	$V_{\theta }:R^{n}\to R$	$V_{\theta }:R^{N}\to R$
Vector fields $f_{\theta }\left(x,t\right)\in T_{x}M$	$f_{\theta }:R^{n}\times R\to R^{n}$	$f_{\theta }^{↑}:R^{N}\times R\to R^{N}$ with tangency constraint [19], optional stabilization [55]
Components of (p,q)-tensor fields $M_{j_{1},\ldots ,j_{q}}^{i_{1},\ldots ,i_{p}}\left(x\right)\in R$	$M_{j_{1},\ldots ,j_{q}}^{i_{1},\ldots ,i_{p}}:R^{n}\to R$	$M_{j_{1},\ldots ,j_{q}}^{i_{1},\ldots ,i_{p}}:R^{N}\to R$, see footnote ^1^

^1^ A tangency constraint on contravariant components of (p,q)-tensors is *not necessarily* required for the vector field $f_{\theta }^{↑}$ to remain tangent to ι(M) and depends on the vector field under investigation.

**Table 3:** Parameterization of scalar functions and tensor components in intrinsic neural ODEs.

Partition of Unity [24,49]	Soft Constraint [18,22]	Pullback [19,21]
Components from all local charts are summed, weighted by a partition of unity.	Function is directly represented in local charts.	Function is pulled back to local chart.
Allows representation of arbitrary smooth functions.	Functions are smooth where charts do not overlap, but are not well-defined at chart transitions.	Allows representation of arbitrary smooth functions.
Differentiating functions generally requires differentiating through chart transition maps, creating computational overload [24].	Chart transition maps do not have to be differentiated.	Chart representations of the embedding ι(M) are differentiated, possibly creating computational overload.

## Data Availability

Not applicable.

## Appendix A. Hamiltonian Dynamics on Lie Groups

We briefly review Hamiltonian systems on manifolds and matrix Lie groups (see also [21], App. A1).

Given a manifold Q with coordinate maps $Q^{i}:Q\to R$ and $p_{i}$ in the basis $dQ^{i}$ on $T_{q}^{*}Q$, we define the symplectic form $\omega \in \Omega^{2}\left(T^{*}M\right)$ as

(A1) $$\omega =dp_{i}\wedge dQ^{i}\;.$$

Let $Y\in X(T^{*}Q)$; then a Hamiltonian $H\in C^{\infty }\left(T^{*}Q,R\right)$ implicitly defines a unique vector field $X_{H}\in X\left(T^{*}Q\right)$ by

(A2) $$dH\left(Y\right)=\omega \left(X_{H},Y\right)\;.$$

In coordinates, $X_{H}$ has the components

(A3) $$\begin{array}{cc} {\dot{q}}^{i} & =\frac{\partial H}{\partial p_{i}}\;, \end{array}$$

(A4) $$\begin{array}{cc} {\dot{p}}_{i} & =-\frac{\partial H}{\partial q^{i}}\;. \end{array}$$

On a Lie group *G*, the group structure allows the identification of $T^{*}G\equiv G\times g^{*}\equiv G\times R^{n}$, e.g., using the pullback ${L_{g}}^{*}:T_{g}^{*}G\to g^{*}$ of the left-translation map $L_{g}:G\to G$, and $\Lambda^{*}:g\to R^{n}$, to define $P_{g}\in R^{n}$ as

(A5) $$P_{g}=\Lambda^{*}L_{g}^{*}P\;.$$

Then the left Hamiltonian $H^{L}:G\times g^{*}\to R$ is defined in terms of $H:T^{*}G\to g$ as

(A6) $$H^{L}\left(g,P_{g}\right)=H\left(g,P)\right)\;.$$

For a matrix Lie group, the left Hamiltonian equations read as follows:

(A7) $$\begin{array}{cc} \dot{g} & =g\Lambda (\frac{\partial H^{L}}{\partial P})\;, \end{array}$$

(A8) $$\begin{array}{cc} \dot{P} & =-d_{g}^{L}H^{L}+{\mathrm{ad}}_{\frac{\partial H^{L}}{\partial P}}^{⊤}P\;, \end{array}$$

with $\Lambda :R^{n}\to g$ as in (13) and $d_{g}^{L}H\in R^{n}$ as in (23).
